# Enhancing flavor and oxidation stability of roasted peanut oil via germination pretreatment

**DOI:** 10.1016/j.fochx.2026.104030

**Published:** 2026-05-25

**Authors:** Gul Nawaz, Wen-ting Yin, Guang-yong Qin, Hua-min Liu, Hui-li Pang, Fan Zhang, Qing-qing Ruan, Zhi Qin, Jun-ru Li

**Affiliations:** a Zhengzhou Research Base, State Key Laboratory of Cotton Bio-Breeding and Integrated Utilization, School of Agricultural and Biomanufacturing, Zhengzhou University, 100 Kexue Avenue, Zhengzhou, 450001, China; b School of Food Science and Technology, Henan University of Technology, 100 Lianhua Road, Zhengzhou 450001, China; c School of Life Sciences, Zhengzhou University, 100 Kexue Avenue, Zhengzhou, 450001, China

**Keywords:** Aroma, Volatile, Sensory, Maillard reaction, Sprouting, Oil processing

## Abstract

This study investigated the effects of short-term peanut germination (0–48 h, 25 °C, 85% relative humidity) prior to microwave roasting (540 W, 8 min) on the flavor and oxidation stability of hydraulic-extracted peanut oil. Extended germination enhanced activities of endogenous enzymes (amylase, lipase, proteases), and increased free amino acids (5.34 → 11.75 mg/g) and soluble sugars (4.69 → 6.12 g/100 g) in peanuts (*p* < 0.05). Increasing germination time increased α- and β-tocopherols, and extended oxidation induction time (9.81 h → 14.89 h) (*p* < 0.05). Increasing germination time increased the relative concentrations of total pyrazines and total phenols, while reducing total aldehydes, leading to enhanced nutty, roasted, butter, and sweet aromas in peanut oil (*p* < 0.05). However, over-germination (48 h) induced undesirable burnt and smoky notes. Appropriate short-term germination can serve as a mild pretreatment to modulate the flavor profile and oxidative stability of roasted peanut oil.

## Graphical abstract

**Figure:**
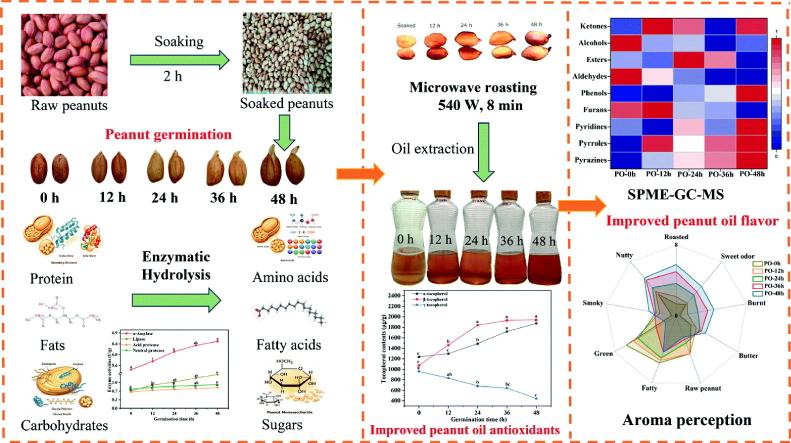

## 1 Introduction

Peanut (*Arachis hypogaea* L.) is a major oil seed crop, cultivated across the globe, especially in China, India, the United States, and Nigeria. Peanuts contain 44–56% oils, 22–30% proteins and many bio-active components including, resveratrol, flavonoids, isoflavones, p-coumaric acid, phytosterols, and phytic acid (Akram et al., 2018; Bilal et al., 2020). Peanuts composition has been associated with various health benefits, containing reducing the risks of cancer, diabetes, coronary heart disease, and respiratory diseases (Adhikari et al., 2018; Akram et al., 2018). Approximately 40% peanuts are produced to peanut oil with an annual production around 6.3 million tons per year. The rich flavor of peanut oil derived from roasted peanut kernels endow it with global consumer appeal, thereby driving the peanut oil processing industry to dedicate substantial efforts toward improving its flavor quality.

The distinctive flavor of peanut oil is developed primarily through traditional hot-air roasting of peanuts before mechanical oil extraction. This process involves several key chemical reactions, including lipid oxidation, caramelization, and the Maillard reaction (Ma et al., 2024). The Maillard reaction is a non-enzymatic browning mechanism initiated by interactions between the amino groups from peptides and amino acids and the carbonyl groups from reducing sugars (Hu et al., 2024). It performs a pivotal function in the formation of key aroma-active constituents in peanut oil, including nitrogen-containing heterocyclic compounds, furans, ketones, and aldehydes (Yin et al., 2022). While the majority of macromolecules, such as proteins and polysaccharides in peanuts, do not directly contribute to the formation of peanut oil flavor, their thermal pyrolysis (above 160 °C) can release small amounts of amino acids and oligosaccharides which serve as flavor precursors (Hu et al., 2021). Although increasing the temperature of hot-air roasting has been shown to enhance peanut oil flavor, this approach is constrained by its low efficiency and severe thermal damage to peanut proteins (Ma et al., 2024). In comparison, microwave roasting features rapid volumetric heating, high energy efficiency, and uniform temperature distribution, thereby shortening processing time, promoting the Maillard reaction and rapid formation of volatile compounds, and reducing thermal denaturation of proteins and the formation of harmful byproducts (Yin et al., 2023; Zhang et al., 2024).

The addition of exogenous enzymes to oilseeds, such as protease, amylase, polygalacturonase, and others, can efficiently degrade macromolecules into small-molecule flavor precursors, and therefore enhance flavor formation during seed roasting (Hu et al., 2025; Ma et al., 2022). However, exogenous enzymes are typically expensive, require substantial modifications to existing oil processing protocols, and are often not permitted by standards in many countries for addition during peanut oil processing. Moreover, peanut oil extracted from roasted peanuts that underwent exogenous enzymatic hydrolysis exhibited notable deviations in flavor characteristics compared to conventional peanut oil. For instance, it may develop an excessive caramel flavor and introduce fermented aromas that are originally absent from conventional peanut oil (Ma et al., 2022).

The utilization of natural endogenous enzymes in peanut oil flavor enhancement may be more economical and technically feasible. During seed germination, water absorption triggers a series of biochemical, physiological, and morphological processes, including organelle remobilization and endogenous enzyme activation (Lu et al., 2023). Consequently, macromolecules such as proteins, starch, cellulose, and triglycerides are hydrolyzed into low-molecular-weight substances, such as peptides, free amino acids, soluble sugars, and free fatty acids, to support early seedling growth (Marti et al., 2020). Numerous studies have demonstrated that germination increases the levels of phenolics, flavonoids, and resveratrol in peanuts, suggesting that germination can be used as an effective natural strategy for nutritional fortification (Adhikari et al., 2018; Yang et al., 2019). One study has reported that germination enhances the DPPH and ABTS radical scavenging activities as well as oxidative stability (oxidation induction time, OIT) of peanut oil (Huh et al., 2025). Nevertheless, to the best of our knowledge, limited research has specifically addressed the effects of germination on the flavor and sensory quality of roasted peanut oil. In particular, the dynamic changes in volatile profiles, sensory characteristics, and oxidative stability of peanut oil across different germination stages remain poorly understood. The present study hypothesizes that appropriate germination as a pretreatment of peanuts can activate endogenous enzymes to promote the accumulation of free amino acids and reducing sugars as critical flavor precursors, thereby enhancing flavor formation in roasted peanut oil.

The primary objective of this study was to evaluate the effects of peanut germination on the activities of key endogenous enzymes and the release of flavor precursors in peanuts, as well as on the flavor profile, sensory attributes, and oxidative stability of the resulting peanut oil. The findings not only provide a practical strategy for improving the flavor quality of peanut oil but also offer novel insights into the application of seed germination in oilseed processing.

## 2 Methods and materials

### 2.1 Ingredients and reagents

Fresh peanuts (variety: Yuanza 6) were harvested in 2024 from Xinxiang City, China. All chemicals employed in the study had a purity exceeding 95%. Petroleum ether, boron trifluoride, hydrochloric acid, citric acid, anhydrous potassium sulfate, copper sulfate, sulfuric acid, phenol, methanol, ethanol, sodium thiosulfate, phenolphthalein, sodium hydroxide (NaOH), sodium hydrogen phosphate, lactic acid, sodium lactate, sodium dihydrogen phosphate, potassium iodide, trichloroacetic acid, and ether were purchased from Kermel Chemical Reagent Co., Ltd. (Tianjin, China). Trichloromethane, and glacial acetic acid were bought from Tianli Chemical Reagent Co., Ltd. (Tianjin, China). Sulfosalicylic acid, Folin-phenol reagent, 3,5-dinitrosalicylic acid (DNS) reagent, α-amylase (α-AL) activity assay kit, all standard aroma compounds, along with 4-nonanol, which served as the internal standard, were bought from Macklin Biochemical Co., Ltd. (Shanghai, China). Casein, isopropanol and hexane were obtained from Sigma-Aldrich (Steinheim, Germany).

### 2.2 Peanut seed germination pretreatment

The germination of peanut was performed on the basis of a published procedure (Yang et al., 2019) with minor modifications. Dehulled peanuts (1000 g) were rinsed and soaked in 3 L purified water (1,3, *w*/*v*) at 25 °C for 2 h. The imbibed peanuts were drained, placed evenly with a spacing of 1 cm × 1 cm in germination trays (30 cm × 23 cm × 4 cm, internal dimensions). Germination was carried out in an LHS-250H automatic climate incubator (Shengyuan Co., Ltd., Qingdao, China) under dark conditions, with the same temperature at 25 °C and 85% relative humidity. The peanuts were manually sprayed with purified water every 6 h to maintain a moist surface environment, with the spray volume controlled to approximately 4 mL per tray each time. The germination process was terminated at 12, 24, 36, or 48 h, respectively, for subsequent sample preparation and analysis. Peanuts subjected to the same soaking process (2 h) without germination treatment were prepared as the control. The non-germinated and germinated peanuts were lyophilized to a moisture of approximately 6.5% in a LGJ-10E freeze dryer at −56 °C (Sihuan Furui Keyi Technology Development Co., Ltd., Beijing, China). After lyophilization, the peanuts were stored temporarily at 4 °C prior to further analysis. The peanut germination rate (%) was calculated as the ratio of germinated peanut seeds (radicle protrusion ≥2 mm) to the total number of samples peanuts (100). The average radicle length of 100 peanut seeds from each germination treatment was measured using a DL91150 Vernier caliper (Deli Group Co., Ltd., Ningbo, China; precision: 0.01 mm). The germination process, germination rate determination, and radicle length measurement were performed in triplicate.

### 2.3 Microwave roasting of peanuts and extraction of peanut oils

For each batch, 250 g of non-germinated or germinated peanuts were microwaved at 540 W and 2.45 GHz for 8 min (Galanz, Co. Ltd., China) while being manually stirred every 2 min. Peanut oils (POs) were extracted from microwaved peanuts using a YKY-6YL-550 machine at a pressure of 60 MPa (Bafang Machinery Co., Ltd., China) according to an established method (Yin et al., 2023). The extracted peanut oil was centrifuged at 4850 ×*g* for 15 min to remove impurities using a LD5–10 centrifuge (Beijing Li Centrifuge Co., Ltd., China). The peanut oils extracted from 0, 12, 24, 36, and 48 h germinated peanuts were named PO-0 h, PO-12 h, PO-24 h, PO-36 h, and PO-48 h, respectively.

### 2.4 Determination of endogenous protease activity in peanuts

The acid and neutral protease activities of peanuts were determined according to an established method (Zhang, Sun, et al., 2023) with a slight modification. Peanut seeds were ground and defatted with 200 mL petroleum ether in a Soxhlet extractor at 45 °C for 6 h. After defatting, the seed meal was dried and passed through a 40-mesh sieve. An aliquot of 1 g peanut meal was suspended in 100 mL of appropriate buffer (lactate buffer, pH 3.0 for acid protease; phosphate buffer, pH 7.2 for neutral protease). The suspension was incubated with magnetic stirring (100 rpm) at 40 °C for 30 min in a water bath, followed by filtration through a filter paper (8 μm pore size). Afterwards, 1 mL of the filtrate was mixed with 1 mL of a 20 g/kg casein solution in a 20 mL centrifuge tube, and incubated at 40 °C for 10 min. The reaction was stopped by adding 2 mL of 0.4 mol/L trichloroacetic acid (TCA), and the mixture was centrifuged at 3040 ×*g* using the LD5–10 centrifuge for 10 min at 25 °C. Thereafter, 1 mL of the supernatant was mixed with 5 mL of 0.4 M Na_2_CO_3_ and 1 mL of Folin-Ciocalteu phenol reagent, followed by incubation at 40 °C for 20 min, and the absorbance was measured at 680 nm using an X-8 UV–vis spectrophotometer (Shanghai Metash Instruments Co., Ltd., China). A calibration curve was constructed using a series of L-tyrosine standards (0.010, 0.020, 0.030, 0.040, and 0.050 g/L) for quantitative analysis.

### 2.5 Determination of endogenous α-amylase activity in peanuts

The α-amylase activity of peanuts was determined using an A930885 α-amylase activity assay kit (Macklin Biochemical Co., Ltd., Shanghai, China) according to the manufacturer's standard method. Briefly, 0.1 g of defatted peanut meal was extracted with 1 mL of distilled water in a 10 mL centrifuge tube. Subsequently, the suspension was incubated at 25 °C for 15 min with magnetic stirring at 100 rpm, then centrifuged at 3040 ×*g* for 10 min at 25 °C. The supernatant was collected and diluted to a final volume of 10 mL with distilled water to prepare the analytic sample (enzyme extract). Thereafter, 0.25 mL of the analytic sample was placed in a centrifuge tube and incubated at 70 °C for 15 min. Subsequently, 0.25 mL starch substrate (reagent 1) was added, and the mixture was incubated at 40 °C for 5 min. The reaction was terminated by the addition of 0.5 mL of 3,5-dinitrosalicylic acid (DNS; reagent 2), and the mixture was heated at 95 °C for 5 min in a water bath. After cooling to 25 °C, the absorbance of the reaction mixture was read at 540 nm using the X-8 UV–vis spectrophotometer. A blank control was conducted following the identical procedure used for the samples, except that 0.25 mL of distilled water was added to replace the starch substrate. The calculation of α-amylase activity was measured according to the protocol provided by the assay kit manufacturer.

### 2.6 Determination of endogenous lipase activity in peanuts

Lipase activity of peanuts was determined using the Chinese national standard method (GB/T 5523–2008). Briefly, 2 g non-germinated or germinated peanuts were ground and homogenized with 1 mL refined peanut oil, 5 mL phosphate buffer (0.05 M, pH 7.4), and 5 mL distilled water. The homogenate was then incubated at 30 °C for 24 h. After incubation, 30 mL of anhydrous ethanol and ether mixture (4,1, *v*/v) was added to the homogenate and thoroughly mixed. The resulting mixture was centrifuged at 3100 ×*g* for 5 min. Subsequently, a 25 mL aliquot of the supernatant was titrated with 0.1 M standard sodium thiosulfate solution. Blank solutions were prepared using the identical procedure, except that the 24-h incubation step was omitted. The endogenous lipase activity (U/g) in peanuts was calculated using Eq. (1).

(1) $$\text{Lipid activity}\;\left(U/g\right)=(V₁–V₀\times C\times 40/m\times \left(100-M\right)\times 40/20\times 100\;$$

Where: *V*₁ and *V*₀ are the titration volume (mL) for sample and blank; *C* denotes the exact concentration (mol/L) of sodium thiosulfate; *m* is the mass (g) of the sample; and M represents the moisture content (%) of the sample.

### 2.7 Determination of crude fat content in peanuts

The total fat content of peanuts was measured by the Soxhlet solvent extraction method. In brief, around 3 g crushed peanuts were subjected to extraction in a Soxhlet extractor using 200 mL petroleum ether at 45 °C for 6 h. Following extraction, the solvent was removed from the fat under vacuum (0.1 MPa gauge pressure) at 45 °C for 10 min using a RV 10 rotary evaporator (IKA-Werke GmbH & Co. KG, Staufen, Germany). The residual oil was oven dried at 105 °C to constant weight, cooled in a desiccator, and weighed to determine crude fat content calculation (expressed as a percentage of dry sample weight).

### 2.8 Determination of crude proteins and free amino acids in peanuts

The protein content of peanuts was quantified using the Kjeldahl method. The total content of free amino acids in peanuts was quantified using a S7130 amino acid analyzer (Syknm, Germany) based on a previous published method (Yang et al., 2024). Approximately 1 g defatted peanut meal was extracted with 50 mL of 0.01 M (HCl) at 25 °C with magnetic stirring (150 rpm) in a water bath for 30 min. The mixture was filtered through a filter paper (8 μm). The obtained (1 mL) of filtrate was mixed with 4% sulfosalicylic acid (1,1, *v*/v) and allowed to stand for 15 min. Subsequently, the sample was centrifuged using a GX16R centrifuge (Hunan Hengnuo Instrument Equipment Co., Ltd., China) at 9728 ×g at 4 °C for 10 min. Finally, the solution was passed through a 0.22 μm polyether sulfone (PES) filter membrane, and 20 μL aliquot of the solution was analyzed using the amino acid analyzer.

### 2.9 Determination of total carbohydrates, reducing sugars, and soluble sugars in peanuts

The total carbohydrate contents of peanuts were quantified using the phenol‑sulfuric acid method, following the procedure reported by Yin et al. (2022). In brief, 1 g defatted peanut meal was hydrolyzed with 25 mL of 1 mol/L hydrochloric acid and heated in a water bath at 95 °C for 40 min. The mixture was filtered through filter paper (8 μm) after cooling to room temperature. Subsequently, 1 mL of the filtrate was mixed with 1 mL of 95% phenol and 5 mL sulfuric acid (H_2_SO_4_), incubated at 25 °C for 40 min. The absorbance of the resulting solution was measured at 490 nm using a UV–Vis spectrophotometer. A blank was prepared without the sample and containing 1 mL of 95% phenol and 5 mL sulfuric acid, with the remaining procedure being identical to that of the sample group. Total carbohydrate content was calculated using Eq. (2):

(2) $$\;\;\text{Total carbohydrate}\;\left(g/100\;g\right)=x\times V_{t}\times D/\left(W\times V_{0}\times 10^{4}\right)\;$$

Where, *x* represents the carbohydrate content (μg) in the sample test solution, which is calculated from the glucose standard curve; *V*_*t*_ is the total volume (mL) of the sample, *D* is the dilution factor (distilled water), *W* is the weight (g) of sample, and *V*_0_ is the test volume (mL).

Approximately 1 g defatted peanut meal was combined with 25 mL of distilled water in a 50 mL centrifuge tube, and the solution was vortexed for 40 min and passed a (8 μm) filter paper. The supernatant (sample extract) was subjected for the analysis of reducing sugar and soluble sugar contents. The reducing sugar content in peanut meal was quantified using the DNS method. Briefly, 1 mL aliquot of the sample extract was diluted 20 times with deionized water and reacted with 2 mL of DNS reagent in a test tube. The mixture was placed at 95 °C for 5 min to allow red-brown color development. Subsequently, cooled to room temperature, diluted with 7 mL of distilled water and thoroughly mixed. The absorbance of the solution was measured at 540 nm using the X-8 UV–vis spectrophotometer. A blank was prepared by replacing the sample solution with 1 mL distilled water, following the same procedure. Reducing sugar concentration in defatted peanut meal was calculated according to Eq. (3):

(3) $$\text{Reducing sugar}\;\left(g/100\;g\right)=x\times V_{t}\times D/\left(m\times V_{0}\times 10^{4}\right)\;$$

Where, *V*_*t*_ is the total volume of the sample extract (25 mL), *D* is the dilution factor (20), *V*_0_ is the volume of extract used in the assay (1 mL), *x* is the mass of sugar content obtained from the standard curve, *m* is the mass (g) of the sample.

The soluble sugar content in peanut was quantified according to the phenol‑sulfuric acid procedure (Aruna & Devindra, 2016) with minor modifications. In detail, 1 mL aliquot of the sample extract was transferred to a glass tube and reacted with 1 mL of 5% phenol solution followed by the addition of 5 mL of 98% (H_2_SO_4_). The mixture was vortexed and then left to stand for 20 min at 25 °C. Subsequently, the absorbance of the solution was measured at 490 nm using the X-8 UV–vis spectrophotometer. A blank sample was treated using the same procedure except that the sample extract was replaced with distilled water. Soluble sugar concentration in defatted peanut meal was calculated according to Eq. (4):

(4) $$\text{Soluble sugar}\;\left(g/100\;g\right)=a\times V_{t}\times D/\left(m\times V_{0}\times 10^{4}\right)$$

Where, *V*_*t*_ is the total volume of the sample extract (25 mL), *D* is the dilution factor (20), *V*_*0*_ is the volume of extract used in the assay (1 mL), and a is the mass of sugar content obtained from the standard curve and m is the mass (g) of the sample.

### 2.10 Headspace solid-phase microextraction of volatile compounds in peanuts oils

Volatile compounds in peanut oil samples were extracted by head-space solid-phase micro extraction (HS-SPME) following a previously reported method (Yin et al., 2022). Briefly, 30 μL of 4-nonanol (1.0 mg/mL in dichloromethane) as the internal standard was added to 3 g of peanut oil sample in a 20 mL sealed glass vial with a Teflon-coated septum. The SPME fiber (2 cm, 50/30 μm) was coated with divinylbenzene/carboxen/polydimethylsiloxane (Supelco Corporation, Charlotte, NC, USA). Each sample was incubated for 20 min at 60 °C before extraction. The SPME fiber was exposed to the headspace of the sample vial allowing for a 50 min extraction at 60 °C. The fiber was then desorbed in the gas chromatograph (GC) injection port at 250 °C for 5 min.

### 2.11 GC–MS analysis of volatile compounds in peanuts oil

The volatile and aroma-active compounds in peanut oils were analyzed using a 7890B gas chromatography (GC) coupled with a mass spectrometry (MS) (Agilent Technologies, Inc., Santa Clara, California, USA) following a previously reported procedure (Hu et al., 2024). Volatile compounds were separated using an HP-5MS capillary column (30 m × 0.25 mm × 0.25 μm; Agilent), with helium as a carrier gas (purity ≥99.999%), 1.8 L/min was set at a flow rate. The GC column temperature was begun at 40 °C for a 5 min hold. Subsequently, raised to 130 °C at a rate of 3 °C/min, hold for 5 min, and then climbed to 250 °C at a rate of 10 °C/min and then hold for 5 min. The temperature of the GC injection port was maintained at 250 °C, the ion source at 230 °C, the quadrupole at 150 °C, and the interface at 280 °C, respectively. Volatile compounds were subjected to MS analysis in electron impact ionization mode at 70 eV, operating in full-scan acquisition (*m*/*z* 33–400, 2.0 scans/s). Volatile compounds were identified by matching their mass spectra with the NIST 20 library database and their retention index (RI). The RI of a volatile compound was obtained by running a series of n-alkanes (C_5_–C_30_). Semi-quantification analysis of volatile compounds was performed using the internal standard method. This approach enables relative comparative analysis among PO samples but is not applicable for accurate quantification.

### 2.12 Calculation of relative odor activity values (ROAVs)

The relative odor activity values (ROAVs) of major volatile flavor compounds in peanut oils were determined based on previous studies (Li et al., 2026; Ruan et al., 2025), according to Eq. (5):

(5) $$\text{ROAV}=\left(Cᵢ\times T\mathrm{ₘₐₓ}\right)/\left(Tᵢ\times C\mathrm{ₘₐₓ}\right)\times 100$$

where *C*ᵢ represent the concentration of a volatile flavor compound and *T*ᵢ represents its odor threshold in the oil matrix. *C*ₘₐₓ and *T*ₘₐₓ correspond to the concentration and odor threshold of the compound with the highest odor activity value. Volatile flavor compounds with ROAVs ≥1 were considered to have substantial contributions to the overall aroma profile of peanut oil.

### 2.13 Sensory evaluation of peanuts oils

Sensory evaluation of peanut oil was conducted according to the established method (Yin et al., 2022). A total of 12 trained panelists (6 females, 6 males; aged 21–26 years) with no known peanut or peanut oil allergies participated in the sensory evaluation. This study adhered to human subject research ethical standards. Ethical approval was not required by the Henan University of Technology, and all participants provided informed consent after being fully briefed on the study details. Rigorous protocols were implemented to protect participants' privacy and rights throughout the process. Each peanut oil sample (5 mL) was transferred into a 75 mL brown tasting glass which was marked with a random 3-digit code. The sampling cup was maintained at 35 °C for 30 min to stabilize the aroma. Each participant smelled and tasted the oil samples and rated nine aroma characteristics such as nutty, roasted, sweet, burnt, butter, raw peanut, fatty, green, and smoky on 10 cm continuous line scales. The panelists' responses were collected using Compusense Cloud software (Compusense Inc., Canada) in individual booths at the Henan University of Technology Sensory Evaluation Centre conforming to ISO: 8589:2007.

### 2.14 Determination of AV, POV, OIT, and color indices of peanut oils

The acid values (AV) of peanut oil samples were measured determined according to the procedures of ISO 660:2020. The peroxide values (POV) of peanut oils were measured according to the procedures of ISO 3960:2017. The oxidation stability of a peanut oil samples was determined as the oxidation induction time (OIT, hours) using a 743 Rancimat instrument (Metrohm AG, Herisau, Switzerland) according to an established procedure (Zhang et al., 2024). About 5 g peanut oil sample was measured at 110 °C with an air flow of 20 L/h.

Color parameters of peanut oil samples were measured with a bench-top spectrophotometer (CS-821 N, Hangzhou Caipu Technology Co., Ltd., China). The CIE Lab color system was applied to obtain L* (lightness), a* (red-green axis), b* (yellow-blue axis), and *ΔE* (total color difference). *ΔE* value was calculated according to Eq. (6) reported by Huh et al. (2025).

(6) $$\mathrm{\Delta E}=\sqrt{{\left(L*-L*0\;\right)}^{2}+{\left(a*-a*0\right)}^{2}+{\left(b*-b*0\right)}^{2}}$$

Where L*_0_, a*_0_, and b*_0_ represent the lightness, redness/greenness, and yellowness/blueness values of the non-germinated peanut oil as the control, respectively.

### 2.15 Determination of tocopherol contents of peanuts oils

The tocopherols in peanut oil were analyzed using high-performance liquid chromatography (HPLC) following the methodology outlined by Zhang et al. (2024). An HPLC system (E2695, Waters, CA, USA) equipped with a fluorescence detector (2475 FLD), was employed to analyze tocopherol content. Briefly, 0.5 g peanut oil was dissolved in 10 mL of n-hexane, and the resulting solution was filtered through a 0.22 μm PTFE membrane before injection into the HPLC system. The HPLC was coupled with a Waters XBridege BEH Amide column (250 mm × 4.6 mm, 5 μm) and maintained at 40 °C, with injection volume of 10 μL was used for each analysis. The mobile phase consisted of a hexane-isopropanol mixture (99:1, *v*/v) delivered at a flow rate of 1 mL/min. The excitation and emission wavelengths were set at 295 nm and 330 nm, respectively.

### 2.16 Statistical analysis

To determine significant differences in volatile flavor components and physicochemical parameters (*p* ≤ 0.05), two-way ANOVA (sample × germination time) followed by Duncan's multiple range test was performed using SPSS Statistics 23 software (IBM Corp., Armonk, NY, USA). For sensory data, two-way ANOVA (sample × panelist) was applied to analyze the main effects of samples, panelists, and sample-panelist interaction, and Tukey's HSD test was used for *post-hoc* multiple comparisons. Germination treatments, preparation of peanut oil samples, and all analytical determinations were conducted in three independent replicates (*n* = 3). Sensory evaluation was performed by 12 trained panelists with three replicates per panelist (*n* = 12 × 3 = 36). All data are presented as mean ± standard deviation (SD). Origin 2021 software (OriginLab Co., USA) was used to plot all figures. Pearson correlation and principal component analysis (PCA) were conducted via Origin Pro 2024 software (Origin Lab Corporation, USA) to examine the relationships between the germinated PO samples and sensory attributes. The data used for principal component analysis (PCA) were the mean sensory ratings averaged from three replicates by 12 trained panelists.

## 3 Result and discussion

### 3.1 Changes in peanut m**orphology** and endogenous enzyme activities during germination

**Fig. 1:**
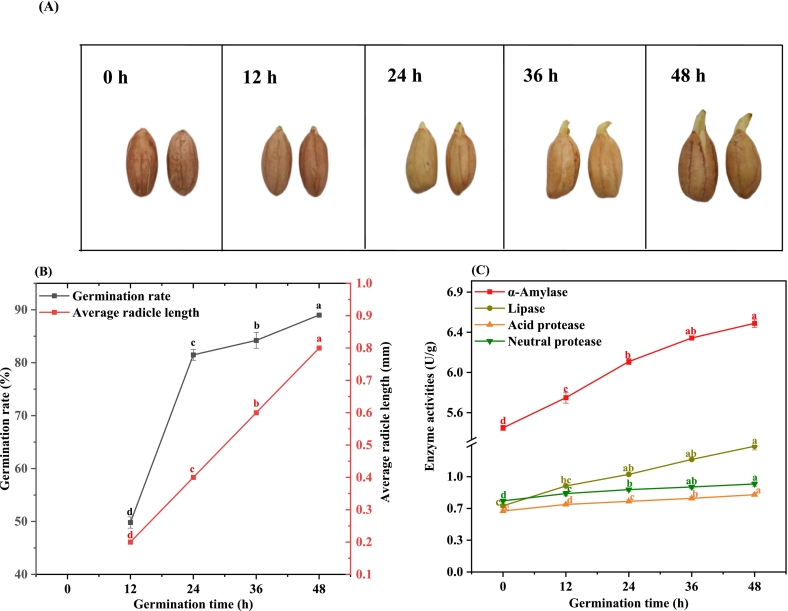
Changes in (A) seed morphology, (B) germination rate and average radicle length, and (C) endogenous enzyme activities of peanuts germinated for different periods.

Peanuts exhibited an increase in volume following imbibition, and the development of radicle was observed after 12 h of germination **(**Fig. 1A**)**. The average radicle length reached 2.11 ± 0.05 mm, 4.25 ± 0.04 mm, 6.85 ± 0.05 mm, and 8.96 ± 0.08 mm, respectively, after 12 h, 24 h, 36 h, and 48 h germination. The germination rate of peanuts increased progressively with germination time, i.e. 49.81% (12*h*), 81.48% (24 h), 84.21% (36 h), and 89.0% (48 h), respectively (*p* < 0.05) **(**Fig. 1B**)**. Notably, the germination rate increased rapidly within the initial 24 h, followed by a slower increasing rate thereafter (*p* < 0.05).

**Fig. 2:**
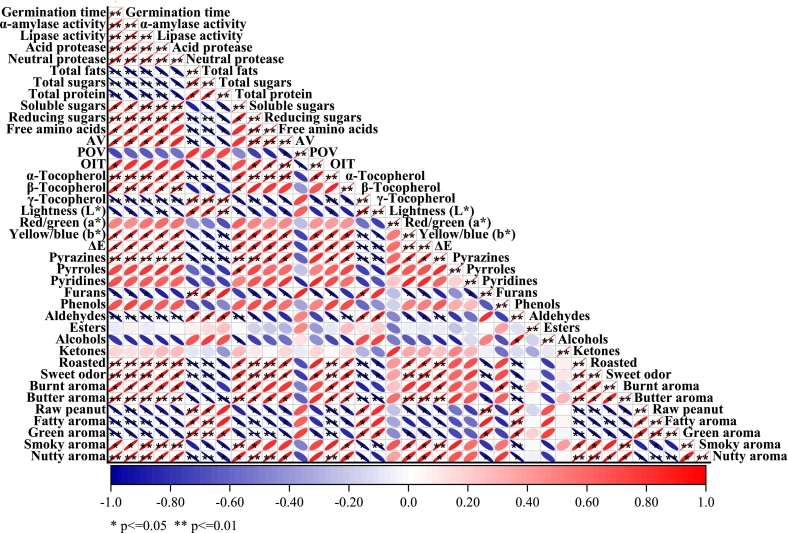
Correlation of germination time, enzymes activities and key compositions in peanuts, aroma-active compounds and sensory profile of peanut oils.

**Fig. 3:**
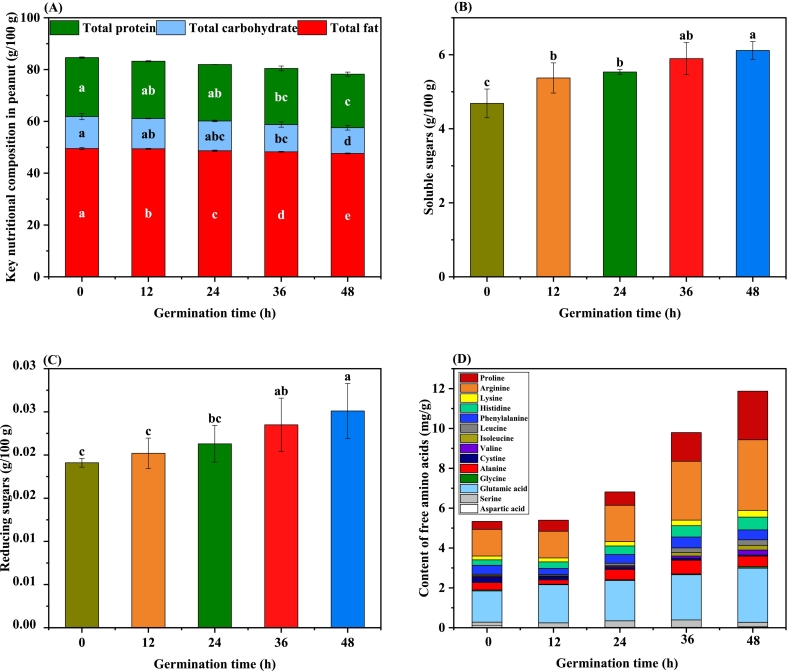
Changes in contents of (A) key nutritional composition, (B) soluble sugars, (C) reducing sugars, (D) free amino acids in peanuts germinated for different periods.

The activities of α-amylase, lipase, and proteases progressively raised with the increasing germination time, as illustrated in Fig. 1C. Pearson correlation analysis **(**Fig. 2**)** showed a strong positive correlation between germination time and enzyme activities (α-amylase, *r* = 0.99; lipase, r = 0.99; acid protease, r = 0.99; and neutral protease, *r* = 0.98) (*p* < 0.05). Specifically, when germination time increased from 0 to 48 h, α-amylase activity increased dramatically from 5.43 to 6.55 U/g, lipase activity increased from 0.71 to 1.35 U/g, acid protease activity increased from 0.65 to 0.83 U/g, and neutral protease activity increased from 0.76 to 0.94 U/g (*p* < 0.05). This suggests that prolonged germination time may enhance activities of multiple endogenous enzymes in peanut seeds. As a result, total fats, total sugars, and total proteins in peanuts were significantly reduced after germination (Fig. 3, *p* < 0.05). After seed imbibition, embryonic cells break dormancy and initiate the synthesis and secret gibberellins (GAs), which diffuse to storage tissues (e.g., cotyledons, aleurone layers) to induce the synthesis of hydrolases, including amylases, proteases, and lipases (Chaudhari et al., 2023; Li et al., 2014). These enzymes then decompose stored substances, including triglycerides, carbohydrates, proteins, etc., to provide both energy and nutrients for hypocotyl elongation and radicle emergence in early germination (Ali and Elozeiri, 2017, Sattar, Ali and Hasnain, 2017).

### 3.2 Changes in key compositions of peanuts during germination

#### 3.2.1 Total fat content

As shown in Fig. 3A, a steady decrease in the total fat content was observed during germination, declining from 49.51 to 47.62 g/100 g (*p* < 0.05). Germination time exhibited a strong negative correlation with total fat content in peanuts (*r* = −0.98, *p* < 0.01). This reduction in fat content is probably attributed to the increased activity of lipase, which catalyzes fat catabolism during germination to provide energy for embryonic growth (Sattar et al., 2017). Similarly, Li et al. (2014) reported a 2–3 fold reduction in the total fat content in various peanut varieties during germination at 25 °C for 5 days. The degradation of carbohydrates and fats supplies the energy required for metabolic activities in seeds, including the formation of enzymes, structural proteins, and other biological molecules (Li et al., 2014).

#### 3.2.2 Total protein content and free amino acids

Fig. 3A**&D** illustrates the effect of germination on the total protein and free amino acid contents in peanut kernels. Increasing germination time from 0 to 48 h progressively reduced the total protein content from 22.76 to 20.65 g/100 g (by 9.27%) (*p* < 0.05). Concurrently, free amino acid content increased significantly from 5.34 mg/g to 11.75 mg/g (*p* < 0.05). Pearson correlation analysis (Fig. 2) showed that germination time exhibited a strong negative association with total protein content (*r* = −0.96) whereas positive association with free amino acid content (*r* = 0.96) in peanut seeds (*p* < 0.01). Germination activates enzymes involved in the hydrolysis of storage proteins, particularly conarachin and arachins, and releases peptides and free amino acids for new protein synthesis and metabolic regulation during seedling development (Rao et al., 2018). A total of 14 amino acids were identified, with glutamic acid and arginine being the most abundant. These findings are consistent with the study of Urbano et al. (2005), which reported that the enhancement in soluble non-protein nitrogen during germination was associated with a reduction in the intensity of the polypeptide bands of seed storage proteins and elevated protease activity in germinated peas. It can be inferred that the partial predigestion of protein during germination, along with enhanced protease activity in the germinated peanut kernels, may play a role in the generation of precursors for volatile compounds through the Maillard reaction in peanut oils.

#### 3.2.3 Total carbohydrates, soluble sugars and reducing sugars

The chemical changes in total carbohydrate, soluble sugars, and reducing sugars during germination are presented in Fig. 3A, B, & C. A significant decreased (*p* < 0.05) was observed in the content of total carbohydrate during the early germination stages, reflecting their utilization as a primary energy source to support metabolic activity and seedling development (Li et al., 2014). Compared with ungerminated peanuts (12.36 g/100 g), the content of total carbohydrate declined continuously with the increasing germination time to 9.96 g/100 g (19.42% reduction) after 48 h germination (*p* < 0.05). The decline in total carbohydrate during germination is closely associated with the activity of hydrolytic enzymes, particularly α-amylase, which catalyzes the breakdown of starch into soluble sugars to support sprout respiratory metabolism and energy availability for growth (Ji et al., 2022). Moreover, the degradation of sugars not only provides a vital energy source for embryonic growth but also facilitates the biosynthesis of structural proteins and other essential metabolic compounds during germination (Li et al., 2014).

Germination time exhibited a strong negative association with total carbohydrate content (*r* = −0.99), whereas a positive association with contents of soluble sugars (*r* = 0.95) (*p* < 0.01). The contents of soluble sugars increased progressively with increasing germination time, from 4.69 g/100 g (0 h) to 6.12 g/100 g (48 h) (*p* < 0.05). Similarly, the concentration of reducing sugars exhibited a continuous increase during germination, from 0.019 g/100 g (0 h) to 0.025 g/100 g (48 h) (*p* < 0.05). This was probably attributed to the accumulation of cellulosic glucose via metabolic activity (Li et al., 2014). The comparable rise in reducing sugars content during germination may be attributed to the enzymatic hydrolysis of residual carbohydrates. The observed increases were primarily attributed to amylase activity, resulting in the formation of sugars (Kim et al., 2021).

### 3.3 Tocopherol contents of peanut oils

**Fig. 4:**
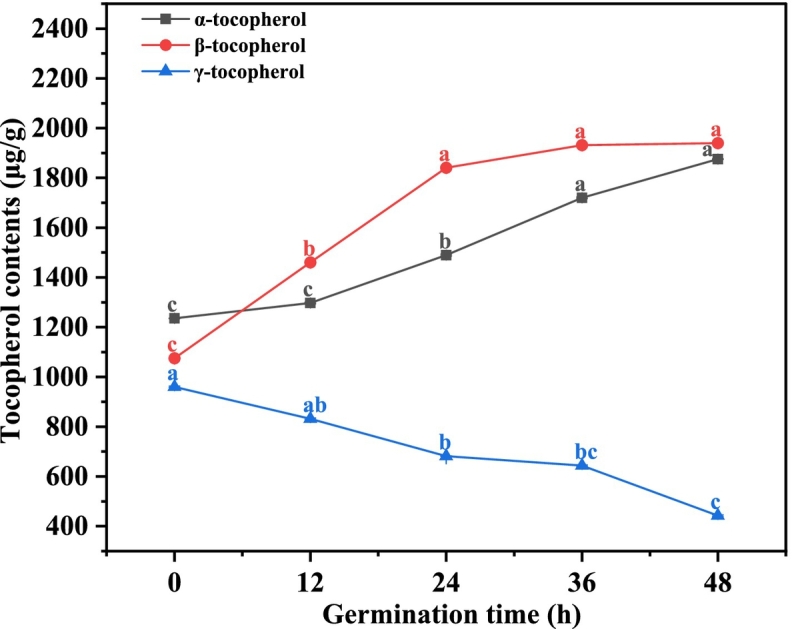
Changes in tocopherol contents in peanut oils extracted from peanuts germinated for different periods.

Tocopherols are important antioxidants naturally present in peanut oil, playing a key role in preventing oxidative degradation and prolonging its shelf life (Idrissi et al., 2024). Three tocopherol isomers (α-, β-, and γ-) were identified in non-germinated and germinated peanut oils (Fig. 4). Germination time showed strong positive correlations with α-tocopherol (*r* = 0.99) and β-tocopherol (*r* = 0.93), while a strong negative correlation with γ-tocopherol (r = −0.99) in peanut oils (*p* < 0.01). Specifically, after 48 h germination, α-tocopherol increased from 1235.58 to 1875.51 μg/g, and β-tocopherol rose from 1074.82 to 1939.50 μg/g, while γ-tocopherol decreased from 960.11 μg/g to 442.39 μg/g after 48 h germination (*p* < 0.05). These findings are aligned with previous studies reported that α-tocopherol increased in quinoa seeds and flaxseeds during germination (Li et al., 2019; Nazih et al., 2025). Zhang et al. (2007) suggested that the decline in γ-tocopherol during germination may be attributed to γ-methyltransferase-mediated interconversion, which catalyzes γ-tocopherol methylation to α-tocopherol, as reported in canola oil. Tocopherols act as radical scavengers by donating hydrogen atoms to neutralize free radicals. Higher concentration of γ-tocopherol contributes more effectively to oil thermal oxidation stability; however, α-tocopherol exhibits the highest biological activity (Nazih et al., 2025).

### 3.4 Effect of germination on oxidation parameters and color of peanut oils

**Fig. 5:**
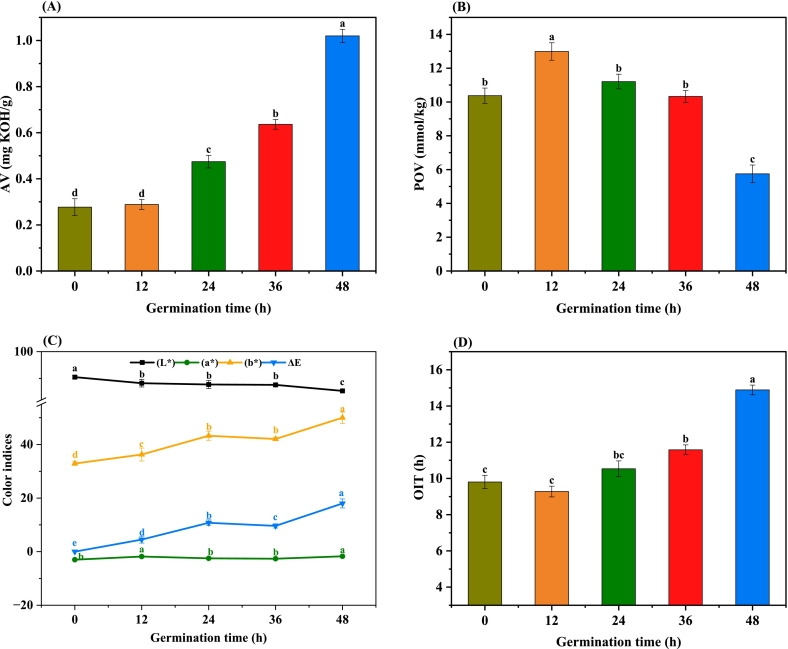
Effect of germination on (A) acid value (AV), (B) peroxide value (POV), (C) color indices, and (D) oxidation induction time (OIT) of peanut oils.

Fig. 5 presents key analytical parameters of the peanut oils, including acid value (AV), peroxide value (POV), oxidation induction time (OIT), and color indices. AV describes the levels of free fatty acids (FFAs) in oil (Pointner et al., 2024). Germination significantly increased the AV of peanut oil (*p* < 0.05), rising from 0.28 mg KOH/g in PO-0 h to 1.02 mg KOH/g in PO-48 h. Zhang et al. (2023) observed a similar rise in AV in flaxseed oil after flaxseed germination. The increase in AV can be associated with enhanced lipolytic enzyme activity during germination, such as lipases and phospholipases, which catalyze the breakdown of triglycerides and phospholipids into free fatty acids (Zhang et al., 2023).

POV reflects the extent of primary lipid oxidation, indicating the concentration of hydroperoxides in oil (Pointner et al., 2024). Compared to oil extracted from non-germinated peanuts (POV = 10.37 mmol/kg), POV increased in PO-12 h (12.99 mmol/kg) (*p* < 0.05). However, with prolonged germination to 48 h, the POV of oils declined progressively to 5.75 mmol/kg (*p* < 0.05). The initial increase in the POV of peanut oil was attributed to hydroperoxide accumulation, promoted by the increased release of free fatty acids during germination (Zhang, Sun, et al., 2023), consistent with the elevated AV. The subsequent decrease in POV with prolonged germination was mainly due to the conversion of unstable hydroperoxides into secondary oxidation products, such as aldehydes and esters, jointly induced by enzymatic oxidation during germination and thermal oxidation during roasting (Pointner et al., 2024).

Germination pretreatment had a significant effect on the color of peanut oil (Fig. 5C). Germination time exhibited a strong negative correlation with the L* value (*r* = −0.94) and positive correlations with the a* value (*r* = 0.58) and b* value (*r* = 0.95) in peanut oils (*p* < 0.01). Specifically, the lightness (L* value) of peanut oil reduced from 90.50 to 85.36 with prolonged germination time (*p* < 0.05), indicating a darker appearance as germination progressed. Concurrently, the b* value (yellowness) increased from 32.90 to 50.01 (*p* < 0.05). In addition, the ΔE value, which reflects the total color differences in PO samples, increased significantly with increasing germination time (*p* < 0.05). The color change in peanut oil may be attributed to the variation in browning pigments generated via the Maillard reaction (Yin et al., 2022).

Oxidation induction time (OIT) reflects the oxidative stability of oils, which is influenced by fatty acid composition, unsaturation degree, and antioxidant content, and serves as an indicator of oil resistance to oxidative degradation and potential shelf life (Huh et al., 2025; Zhang et al., 2024). The OIT of peanut oils increased from 9.81 h in non-germinated samples to 14.89 h after 48 h of germination (Fig. 5D, *p* < 0.05), showing a gradual increase in oxidative stability as germination time prolonged. As shown in Fig. 2, peanut oil OIT was positively correlated with germination time (*r* = 0.89, *p* < 0.05), seed reducing sugars (*r* = 0.91, *p* < 0.05), free amino acids (r = 0.95, *p* < 0.05), α-tocopherol (*r* = 0.92, *p* < 0.05), pyrazines in oil (*r* = 0.83, *p* < 0.05), b* index (r = 0.89, *p* < 0.05), Δ*E* (*r* = 0.88, *p* < 0.05), and peroxide value (POV, *r* = 0.95, *p* < 0.05) of peanut oil. In contrast, OIT was negatively correlated with furans (*r* = −0.92, *p* < 0.05), aldehydes (*r* = −0.74, *p* < 0.05), γ-tocopherol (*r* = −0.90, *p* < 0.05), L* index (*r* = −0.84, *p* < 0.05), and acid value (AV, *r* = −0.99, *p* < 0.05) of peanut oil. The observed correlation trends indicate that prolonged germination is closely linked to changes in Maillard reaction substrates, which may be associated with the accumulation of antioxidant-related Maillard-derived compounds such as pyrazines and browning pigments (Ruan et al., 2025). Meanwhile, the elevated acid value and lipid oxidation level observed during germination may also present potential unfavorable impacts on oil oxidative stability.

### 3.5 Effect of germination on volatile composition of peanut oils

**Fig. 6:**
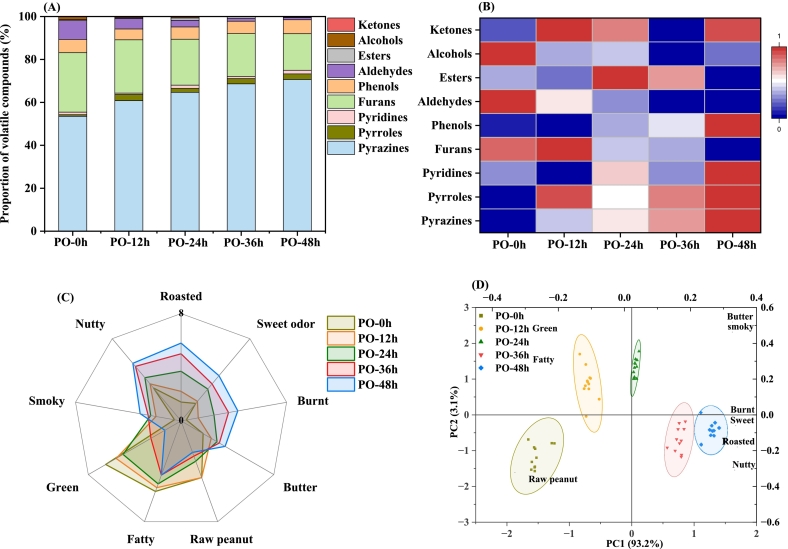
**(**A) Relative proportion of volatile compounds, (B) heat map visualized by *Z*-score normalization for aroma-active compounds, (C) sensory aroma profile, and (D) principal component analysis of sensory characterization of peanut oils.

Volatile compounds in peanut oils varied with germination time (Fig. 6A). PO-0 h contained 53.50% pyrazines, 27.68% furans, 8.79% aldehydes, 1.22% pyridines, 0.76% pyrroles, 6.14% phenols, 1.40% alcohols, 0.47% esters, and 0.04% ketones. As germination time increased, the proportions of pyrazines and phenolic compounds increased gradually, whereas those of furans and aldehydes decreased significantly (*p* < 0.05). As shown in Table 1, a total of 63 distinct potential aroma-active compounds were detected in peanut oils, including 19 pyrazines, 6 pyrroles, 6 pyridines, 6 furans, 4 phenols, 12 aldehydes, 4 esters, 4 alcohols, and 2 ketones. The relative concentrations of different compound classes were visually compared in a heat map obtained through z-score normalization in Fig. 6B. Among them, 19 major volatile flavor compounds with ROAVs ≥1 in peanut oil are summarized in Table 2.

**Table 1:** Effect of peanut germination pretreatment on volatile flavor compounds of peanut oils (POs).

RI A	Volatile flavor compounds B	Odor description C	Concentration (mg/kg) D				
			PO-0 h	PO-12 h	PO-24 h	PO-36 h	PO-48 h
812	2-Methylpyrazine	nutty, roasted	12.85 ± 1.59^a^	13.76 ± 0.51^a^	10.68 ± 0.04^a^	12.50 ± 0.30^a^	13.60 ± 0.13^a^
901	2,5-Dimethylpyrazine	nutty, roasted	19.99 ± 0.00^d^	29.35 ± 0.86^b^	26.30 ± 0.02^c^	29.87 ± 0.51^b^	32.60 ± 0.37^a^
908	2,3-Dimethylpyrazine	popcorn, roasted	ND	ND	4.62 ± 0.00^c^	5.33 ± 0.24^b^	6.08 ± 0.00^a^
919	Ethenylpyrazine	popcorn, buttery	ND	0.39 ± 0.02^a^	ND	ND	ND
987	2-Ethyl-6-methylpyrazine	roasted, nutty, fruity	1.66 ± 0.07^e^	2.79 ± 0.10^d^	3.30 ± 0.02^c^	3.81 ± 0.12^b^	4.20 ± 0.05^a^
991	Trimethylpyrazine	roast, potato	8.81 ± 0.44^d^	13.21 ± 0.37^c^	13.30 ± 0.01^c^	14.96 ± 0.27^b^	16.02 ± 0.09^a^
1005	2-Ethenyl-6-methylpyrazine	burn, roasted	ND	ND	0.57 ± 0.00^b^	0.68 ± 0.00^a^	ND
1008	Isopropenylpyrazine	burnt	0.42 ± 0.00^a^	ND	ND	ND	ND
1010	Pyrazinamide	popcorn	ND	ND	ND	1.47 ± 0.0^a^	1.62 ± 0.00^a^
1012	Acetylpyrazine	nutty, popcorn	ND	0.73 ± 0.02^a^	ND	ND	ND
1069	3-Ethyl-2,5-dimethylpyrazine	roasted, sweet	4.66 ± 0.54^a^	3.41 ± 2.96^a^	8.30 ± 0.02^a^	8.15 ± 0.06^a^	8.52 ± 0.07^a^
1073	2,6-Diethylpyrazine	roasted, roasted	0.91 ± 0.10^a^	0.87 ± 0.41^a^	1.31 ± 0.01^a^	1.08 ± 0.42^a^	1.56 ± 0.02^a^
1076	2,3-Dimethyl-5-ethylpyrazine	roasted	ND	ND	1.32 ± 0.00^a^	ND	1.57 ± 0.00^a^
1088	2-Methyl-6-(1-propenyl) pyrazine	sweet, nutty	ND	ND	0.96 ± 0.00^a^	1.01 ± 0.00^a^	1.06 ± 0.00^a^
1103	1-(5-Methylpyrazin-2-yl)ethan-1-one	roasted, popcorn	ND	0.25 ± 0.03^a^	0.19 ± 0.00^b^	0.23 ± 0.01^ab^	0.23 ± 0.00^ab^
1107	1-(6-Methyl-2-pyrazinyl)-1-ethanone	roasted, sweet	ND	ND	ND	ND	0.35 ± 0.00^a^
1121	Pyrazine	nutty, popcorn	0.11 ± 0.00^a^	ND	ND	ND	ND
1126	6,7-Dihydro-5-methyl-5H-cyclopenta[*b*]pyrazine	popcorn	0.38 ± 0.09^a^	0.45 ± 0.05^a^	0.43 ± 0.00^a^	0.55 ± 0.02^a^	0.55 ± 0.00^a^
1147	3,5-Diethyl-2-methylpyrazine	roasted peanut	0.54 ± 0.0^a^	0.60 ± 0.07^a^	0.50 ± 0.35^a^	0.59 ± 0.42^a^	0.90 ± 0.06^a^
	**Sum of pyrazines**		**50.32** **±** **2.84**^**d**^	**65.82** **±** **5.38**^**c**^	**71.80** **±** **0.48**^**bc**^	**80.22** **±** **2.38**^**ab**^	**88.87** **±** **0.79**^**a**^
713	2-Methyl-1H-pyrrole	smoky, nutty	0.18 ± 0.06^a^	0.06 ± 0.04^a^	0.16 ± 0.02^a^	0.21 ± 0.04^a^	0.16 ± 0.07^a^
895	2,3-Dimethyl-1H-pyrrole	roasted	ND	ND	ND	ND	0.19 ± 0.00^a^
898	3-Methyl-1H-Pyrrole	sweet, nutty	ND	1.31 ± 0.00^a^	1.53 ± 0.00^a^	1.39 ± 0.00^a^	1.75 ± 0.00^a^
1006	1H-pyrrole-2-carbaldehyde	almond, popcorn	ND	1.30 ± 0.00^a^	ND	0.63 ± 0.03^c^	0.67 ± 0.00^b^
1122	1-Methylpyrrole-2-carboxaldehyde	salty, roasted	0.43 ± 0.07^a^	0.44 ± 0.10^a^	0.38 ± 0.02^a^	0.52 ± 0.10^a^	0.46 ± 0.00^a^
1269	4-Formyl-3,5-dimethyl-1H-pyrrole-2‑carbonitrile	roasted peanut	0.10 ± 0.02^a^	ND	0.06 ± 0.00	0.09 ± 0.02^b^	0.08 ± 0.0^ab^
	**Sum of pyrroles**		**0.72** **±** **0.15**^**d**^	**3.12** **±** **0.13**^**ab**^	**2.13** **±** **0.04**^**c**^	**2.83** **±** **0.18**^**b**^	**3.30** **±** **0.08**^**a**^
1013	1-Methyl-1,4-dihydropyridine-3‑carbonitrile	peanut, milk	0.33 ± 0.20^a^	0.12 ± 0.00^a^	0.73 ± 0.00^a^	0.42 ± 0.27^a^	0.86 ± 0.00^a^
1022	1-(2-pyridinyl)-Ethanone	popcorn, must	ND	ND	ND	ND	0.19 ± 0.00^a^
1037	1-(3,4,5,6-tetrahydropyridin-2-yl)ethan-1-one	roasted, bread	ND	ND	0.06 ± 0.00^c^	0.18 ± 0.00^a^	0.17 ± 0.00^b^
1176	1-Acetyl-1,2,3,4-tetrahydropyridine	caramel, nutty	0.17 ± 0.04^a^	0.30 ± 0.05^a^	0.22 ± 0.01^a^	0.23 ± 0.02^a^	0.22 ± 0.00^a^
1183	2-propylpyridine	caramel, roasted	0.65 ± 0.00^a^	ND	0.33 ± 0.00^c^	0.31 ± 0.00^d^	0.34 ± 0.00^b^
1185	2-Pentylpyridine	greasy	ND	0.30 ± 0.04^a^	0.31 ± 0.00^a^	ND	0.34 ± 0.00^a^
	**Sum of pyridines**		**1.15** **±** **0.23**^**bc**^	**0.71** **±** **0.10**^**c**^	**1.65** **±** **0.01**^**ab**^	**1.14** **±** **0.29**^**bc**^	**2.13** **±** **0.01**^**a**^
822	Furfural	nutty	4.37 ± 0.57^a^	5.20 ± 0.14^a^	2.63 ± 0.03^b^	2.14 ± 0.06^b^	2.0 ± 0.00^b^
902	2-Pentanoylfuran	roasted, butter	0.36 ± 00^a^	0.71 ± 0.00^a^	0.78 ± 0.03^a^	1.05 ± 0.11^a^	1.04 ± 0.03^a^
954	5-Methylfurfural	popcorn, bread	1.76 ± 0.18^b^	3.02 ± 0.05^a^	1.83 ± 0.00^b^	1.79 ± 0.04^b^	1.64 ± 0.06^b^
983	2-Pentylfuran	green, fruity	0.63 ± 0.00^d^	1.23 ± 0.05^a^	0.88 ± 0.00^c^	1.13 ± 0.05^ab^	1.01 ± 0.03^b^
1065	Furaneol	sweet, caramel	ND	ND	0.44 ± 0.00^a^	ND	ND
1214	2,3-Dihydrobenzofuran	popcorn, nutty	18.91 ± 1.22^a^	16.70 ± 4.03^a^	17.17 ± 1.51^a^	17.27 ± 1.21^a^	15.66 ± 2.10^a^
	**Sum of furans**		**26.04** **±** **1.97**^**a**^	**26.86** **±** **4.27**^**a**^	**23.73** **±** **1.58**^**b**^	**23.38** **±** **1.46**^**b**^	**21.35** **±** **2.21**^**c**^
1093	2,4-Di-tertbutylphenol	burnt	ND	0.10 ± 0.00	ND	2.68 ± 0.00	ND
1101	Maltol	smoky, burnt	ND	1.09 ± 0.17^ab^	1.04 ± 0.01^b^	1.41 ± 0.08^ab^	1.49 ± 0.17^a^
1301	5-Ethenyl-2-methoxyphenol	burnt wood, nutty	ND	2.53 ± 0.67^a^	2.79 ± 0.00^a^	ND	2.21 ± 0.00^a^
1315	2-Methoxy-4-vinylphenol	spice, clove	5.78 ± 0.17^a^	1.86 ± 0.00^d^	2.57 ± 0.22^c^	2.68 ± 0.14^c^	4.52 ± 0.00^b^
	**Sum of phenolics**		**5.78** **±** **0.17**^**bc**^	**5.58** **±** **0.84**^**c**^	**6.40** **±** **0.23**^**bc**^	**6.77** **±** **0.22**^**b**^	**8.22** **±** **0.17**^**a**^
887	Hexanal	grass, tallow, fat	1.13 ± 0.06^b^	1.60 ± 0.08^a^	0.81 ± 0.04^c^	0.66 ± 0.04^c^	0.64 ± 0.02^c^
894	Heptanal	fat, citrus, rancid	ND	0.12 ± 0.00^a^	ND	ND	ND
947	*(E)-*2-Heptenal	chemical, fatty	ND	0.52 ± 0.00^a^	0.38 ± 0.00^a^	ND	ND
1019	5-Ethyl-1-cyclopentene-1-carboxaldehyde	oily	ND	ND	0.10 ± 0.01^a^	0.10 ± 0.00^a^	ND
1032	Benzeneacetaldehyde	flower, sweet	4.30 ± 0.25^a^	1.38 ± 0.04^b^	1.01 ± 0.06^b^	0.25 ± 0.01^c^	0.20 ± 0.05^c^
1049	(*E*)-2-Octenal	green, fruity	0.19 ± 0.01^b^	0.29 ± 0.01^a^	0.16 ± 0.00^c^	0.09 ± 0.00^d^	ND
1095	Nonanal	fat, citrus, green	0.42 ± 0.03^b^	0.62 ± 0.02^a^	0.33 ± 0.01^c^	0.21 ± 0.02^d^	0.21 ± 0.02^d^
1150	(*E*)-2-Nonenal	green, harsh	ND	0.06 ± 0.00^a^	ND	ND	ND
1251	*(E)*-2-Decenal	tallow, soapy, fatty	0.23 ± 0.01^a^	0.08 ± 0.02^b^	ND	ND	ND
1262	α-Methylbenzyl alcohol	flower, rose	0.36 ± 0.01^a^	0.20 ± 0.04^b^	0.23 ± 0.01^b^	0.17 ± 0.01^b^	0.20 ± 0.04^b^
1304	(*E, E*)-2,4-Decadienal	rancid, fatty, waxy	1.54 ± 0.07^a^	0.37 ± 0.28^a^	0.39 ± 0.00^a^	ND	ND
1352	2-Undecenal	sweet	0.10 ± 0.01^a^	0.02 ± 0.01^a^	ND	ND	ND
	**Sum of aldehydes**		**8.27** **±** **0.45**^**a**^	**5.26** **±** **0.49**^**b**^	**3.44** **±** **0.14**^**c**^	**1.52** **±** **0.08**^**d**^	**1.27** **±** **0.14**^**d**^
808	Ethyl 4-ethoxy-2-oxobutyrate	fruity, harsh	0.08 ± 0.00^a^	0.10 ± 0.00^a^	ND	0.60 ± 0.00^a^	0.04 ± 0.00
1001	Methyl formate	fruity	ND	ND	1.23 ± 0.00^a^	ND	ND
1061	Octyl formate	orange	ND	ND	ND	0.09 ± 0.00	ND
1127	Methyl nicotinate	popcorn	0.36 ± 0.00^a^	0.23 ± 0.0^a^	ND	0.24 ± 0.00^a^	ND
	**Sum of esters**		**0.44** **±** **0.00**^**b**^	**0.32** **±** **0.00**^**bc**^	**1.23** **±** **0.00**^**a**^	**0.93** **±** **0.00**^**a**^	0.04 ± 0.00
745	1-Pentanol	balsamic	0.21 ± 0.00^a^	ND	0.13 ± 0.01^b^	ND	ND
862	1-Hexanol	resin, flower, green	0.73 ± 0.09	ND	ND	ND	ND
973	1-Octen-3-ol	mushroom	0.29 ± 0.03^b^	0.41 ± 0.02^a^	0.46 ± 0.00^a^	ND	0.41 ± 0.01^a^
1067	1-Octanol	waxy, green	0.09 ± 0.00b	0.10 ± 0.00^a^	ND	0.09 ± 0.00^b^	ND
	**Sum of alcohols**		**1.32** **±** **0.13**^**a**^	**0.51** **±** **0.02**^**bc**^	**0.59** **±** **0.01**^**b**^	**0.09** **±** **0.00**^**d**^	**0.41** **±** **0.01**^**c**^
830	2-Butanone	creamy, sweet	0.03 ± 0.00^a^	ND	ND	ND	ND
882	2-Heptanone	garlic, soap	ND	0.17 ± 0.00^a^	0.14 ± 0.00^a^	ND	0.16 ± 0.00^a^
	**Sum of ketones**		**0.03** **±** **0.00**^d^	**0.17** **±** **0.00**^a^	**0.14** **±** **0.00**^c^	**ND**	**0.16** **±** **0.00**^b^

Note: ND indicates not detected; PO-0 h, PO-12 h, PO-24 h, PO-36 h, and PO-48 h represents peanut oil obtained from different times of peanut germination. For each row, values without any same superscript letter are significantly different (*p* < 0.05).

A : RI is the retention index of volatile flavor compounds obtained on the HP-5MS column.

B : Volatile flavor compounds were analyzed by HS-SPME-GC–MS.

C : Odor descriptions of volatile flavor compounds in peanut oil were obtained from Yin et al. (2021), Yin et al. (2022), Yin et al. (2023), Hu et al. (2023), Hu et al. (2024), Zhang et al. (2024), Hu et al. (2025), Xu et al. (2025), and Ruan et al. (2025).

D : Volatile flavor compounds were semi-quantified using the internal standard method.

**Table 2:** Relative odor activity values (ROAV) of important volatile flavor compounds in peanut oil.

Aroma-active compounds A	Odor threshold B (μg/kg)	ROAV C				
		PO-0 h	PO-12 h	PO-24 h	PO-36 h	PO-48 h
2-Methylpyrazine	200	34.45	47.66	16.08	19.96	19.17
2,5-Dimethylpyrazine	2000	5.36	10.16	3.96	4.78	4.58
2,6-Diethylpyrazine	6	80.96	100	65.94	76.41	55.29
3-Ethyl-2,5-dimethylpyrazine	25	100	94.48	100	100	100
Trimethylpyrazine	290	16.29	31.55	13.81	16.21	15.82
2,3-Dimethyl-5-ethylpyrazine	200	0	0	1.99	0	2.41
2,3-Dihydrobenzofuran	500	20.29	23.13	10.34	10.13	9.61
2-Pentylfuran	130	2.61	6.56	2.04	2.56	2.39
Furfural	700	3.34	5.14	1.13	0.90	0.88
2-Methoxy-4-vinylphenol	50	61.98	25.72	15.48	15.75	27.76
5-Ethenyl-2-methoxyphenol	50	0	34.99	16.79	0	13.54
Maltol	210	0	3.61	1.49	1.97	2.17
Benzeneacetaldehyde	154	14.97	6.20	1.98	0.48	0.40
(*E*)-2-heptenal	50	0	7.23	2.26	0	0
(*E*)-2-octenal	4	26.02	49.74	11.77	6.79	0
(*E*)-2-decenal	10	12.31	5.42	0	0	0
Hexanal	73	8.27	15.18	3.34	2.64	2.69
(*E, E*)-2,4-Decadienal	180	4.57	1.43	0.66	0	0
1-Octen-3-ol	36	4.31	7.85	3.85	0	3.46

A : Important volatile flavor compounds in peanut oil are defined by ROAVs ≥1.

B : Odor thresholds in the oil matrix are from Yin et al. (2021), Yin et al. (2022), Hu et al. (2023), Xu et al. (2025), and Ruan et al. (2025).

C : ROAV represents relative odor activity value.

#### 3.5.1 N-heterocyclic compounds

Nitrogen-containing heterocyclic compounds, including pyrazines, pyrroles, and pyridines, are formed during the Maillard reaction and are key contributors to the roasted, nutty, and popcorn-like aromas of peanut oil (Ma et al., 2024; Yin et al., 2022). Pyrazines were identified as the most abundant volatile compounds in peanut oil, and their total concentration increased continuously from 50.32 ± 2.84 to 88.87 ± 0.79 mg/kg as germination time increased (*p* < 0.05). Germination time exhibited strong positive correlations with total pyrazines (*r* = 0.99, *p* < 0.05) and key sensory attributes including nutty aroma (*r* = 0.98, *p* < 0.05), roasted aroma (r = 0.99, *p* < 0.05), and sweet aroma (r = 0.98, *p* < 0.05) in peanut oils. This increase is likely attributed to germination-induced release of soluble sugars and free amino acids (Fig. 2), which provided more precursors for pyrazine formation during roasting. Among the 19 identified pyrazines, several were particularly important based on their relative odor activity values (ROAVs ≥1). Specifically, 2-methylpyrazine (ROAV = 47.66), 2,5-dimethylpyrazine (ROAV = 10.16), 2,6-diethylpyrazine (ROAV = 100), and trimethylpyrazine (ROAV = 31.55) were important aroma contributors in PO-12 h, while 3-ethyl-2,5-dimethylpyrazine (ROAV = 94.48–100) was an important aroma contributor in all PO samples. 2,3-Dimethyl-5-ethylpyrazine was exclusively detected in PO-24 h (ROAV = 1.99) and PO-48 h (ROAV = 2.41). 2,5-Dimethylpyrazine, 2-ethyl-6-methylpyrazine, and 2,3,5-trimethylpyrazine have been identified as key aroma-active compounds in roasted peanut oil, contributing significantly to its characteristic nutty and roasted peanut aromas (Ma et al., 2024; Yin et al., 2022).

#### 3.5.2 O-heterocyclic compounds (furans)

Furans, oxygen-containing heterocylics, are typically generated via the Maillard reaction, caramelization, and lipid oxidation, contributing to caramel, sweet, and roasted aromas in peanut oil (Yin et al., 2022). A total of 7 furans were identified, and their total concentration was the highest in PO-0 h (26.04 ± 1.97 mg/kg) and PO-12 h (26.86 ± 4.27 mg/kg), then gradually decreased to 21.35 ± 2.21 in PO-48 h with prolonged germination time (*p* < 0.05). Germination time exhibited a strong negative correlation with total furan content (*r* = −0.99, *p* < 0.05) in peanut oils. In all PO samples, 2,3-dihydrobenzofuran (nutty and popcorn notes, ROAV = 9.61–23.13) and 2-pentylfuran (earthy and fruity, ROAV = 2.04–6.56) were identified as key aroma-active furans. 2,3-Dihydrobenzofuran mainly originates from sugar degradation and the Maillard reaction (Zhang, Shen, et al., 2023), whereas 2-pentylfuran is primarily formed via the oxidation of linoleic acid (Hu et al., 2024). Furfural was an important volatile flavor compound in PO-0 h, PO-12 h, and PO-24 h, with ROAVs ranging from 1.13 to 5.14. However, it has insignificant contribution to the overall aroma of PO-36 h and PO-48 h, with ROAVs lower than 1.

#### 3.5.3 Volatile phenolics

A total of four volatile phenolic compounds were detected in peanut oils, contributing to burnt and smoky aromas. Overall, the total phenolic content increased with increasing germination time from 5.78 ± 0.17 mg/kg in PO-0 h to 8.22 ± 0.17 mg/kg in PO-48 h (*p* < 0.05). Germination time was positively correlated with total volatile phenolic content (*r* = 0.70, *p* < 0.01), perceived burnt aroma (*r* = 0.93, *p* < 0.01), and smoky aroma (*r* = 0.94, *p* < 0.01). As shown in Tables 2, 2-methoxy-4-vinylphenol was identified as an important volatile phenolic compound in all PO samples, with ROAVs ranging from 15.41 to 61.98. 5-Ethenyl-2-methoxyphenol was absent in PO-0 h and PO-36 h, but was an important volatile phenolic compound in PO-12 h (ROAV = 34.99), PO-24 h (ROAV = 16.79), and PO-48 h (ROAV = 13.54). Maltol was absent in PO-0 h but an important volatile flavor compound in PO-12 h, PO-24 h, PO-36 h, and PO-48 h with ROAVs ranging from 1.49 to 3.61. Presumably, germination promotes the release of bound phenolic compounds via enzymatic degradation of intracellular structures (Kumar et al., 2020), which subsequently contributes to the synthesis of volatile phenolics such as 5-ethenyl-2-methoxyphenol and maltol during roasting of peanut seeds (Zhang et al., 2024).

#### 3.5.4 Aldehydes, ketones, alcohols and esters

Aldehydes, ketones, alcohols, and esters are primarily formed via lipid degradation, caramelization, and the Maillard reaction, contributing to fatty, green, butter, and sweet aromas (Yin et al., 2022). A total of 12 aldehydes were identified in peanut oils. Total aldehyde content was most abundant in PO-0 h (8.27 ± 0.45 mg/kg), and declined continuously with prolonged germination, falling to 1.27 ± 0.14 mg/kg in PO-48 h (*p* < 0.05). Germination time exhibited a strong negative correlation with total aldehyde content (*r* = −0.97, *p* < 0.01) in peanut oils. Most identified aldehydes are lipid oxidation products, including hexanal, (*E,E*)-2,4-decadienal, (*E*)-2-decenal, (*E*)-2-octenal, and (*E*)-2-heptenal (Arsa & Theerakulkait, 2018). Presumably, germination enhanced oxidation stability of peanut oil (Fig. 4, OIT), thereby reducing the formation of lipid-derived aldehydes. Benzeneacetaldehyde (sweet and floral aromas), which originated from the Strecker degradation of phenylalanine (Ma et al., 2024), was most abundant in PO-0 h and decreased as germination time increased (*p* < 0.05). Benzeneacetaldehyde was an important volatile flavor compound in PO-0 h, PO-12 h, and PO-24 h with ROAVs ranging from 14.97 to 1.98. (*E*)-2-Heptenal was an important volatile flavor compound in PO-12 h and PO-24 h with ROAVs of 7.23 and 2.26, respectively. (*E*)-2-Octenal was important to the overall aroma in all PO samples (ROAVs ranging from 6.79 to 26.02), except for PO-48 h (not detected). Hexanal (green and fatty aromas) played an important role in the overall aroma of all PO samples with ROAVs ranging from 2.64 to 15.18. (*E*)-2-Decenal was important to the overall aroma of PO-0 h and PO-12 h with ROAVs of 12.31 and 5.42, respectively. (*E,E*)-2,4-Decadienal was detected in PO-0 h, PO-12 h, and PO-24 h, with corresponding ROAVs of 4.57, 1.43, and 0.66.

Two ketones were identified in peanut oil, with distinct contributions to buttery, fruity, and sweet aromas (Yin et al., 2022). Specifically, 2-heptanone is formed via linoleic acid oxidative degradation (Ruan et al., 2025). It was detected only in PO-12 h, PO-24 h, and PO-48 h, but it was not a key aroma-active compound (ROAV <1). 2-Butanone was detected exclusively in non-germinated peanut oil (PO-0 h) and is generated through lipid oxidation (Hu et al., 2024). Four alcohols were detected in peanut oils, and their total concentration was higher in PO-0 h (1.3 mg/kg) than in germinated PO samples (*p* < 0.05). Specifically, 1-hexanol was detected only in PO-0 h, while 1-octen-3-ol was found in all PO samples except PO-36 h, with ROAVs ranging from 3.46 to 7.85, both of which are lipid degradation products (Xu et al., 2025). Other alcohols had a minor impact (ROAVs <1) on the overall aroma of peanut oil due to their high odor thresholds. Esters are either natural volatile compounds or products of lipid oxidation in oilseeds (Zhang et al., 2024). Four esters were identified in peanut oils, which also had a minor impact (ROAVs <1) on overall aroma of peanut oil.

### 3.6 Effect of germination on sensory aroma profile of peanut oils

The aroma characteristics of peanut oils are presented in Fig. 6C. Significant differences in all sensory attributes were observed among POs (*p* < 0.05), with no significant sample-panelist interaction, confirming the panel's reliability. As shown in Fig. 6D, principal component analysis (PCA) clearly differentiated the sensory profiles of POs from different germination periods. The first two principal components explained 95.7% of the total variance (PC1: 92.6%; PC2: 3.1%), and the distinct separation of samples in the score plot demonstrated that germination time significantly altered sensory characteristics of peanut oils.

The sensory profile of non-germinated peanut oil (PO-0 h) was dominated by strong green note (6.5 ± 0.4) and fatty note (5.6 ± 0.3), along with moderate raw peanut (4.5 ± 0.3) and roasted aromas (4.5 ± 0.1), as well as weak nutty (3.2 ± 0.2), butter (1.9 ± 0.1), sweet (1.7 ± 0.3), smoky (0.5 ± 0.3), and burnt notes (0.4 ± 0.1). With increasing germination time, the perceived intensities of nutty, roasted, butter, sweet, burnt, and smoky notes in peanut oil (POs) significantly increased (*p* < 0.05), whereas green and fatty notes decreased progressively (*p* < 0.05). Notably, intensified nutty, roasted, buttery, and sweet notes are desirable sensory properties for peanut oil, while pronounced burnt and smoky notes are typically undesirable (Yin et al., 2022). Prolonged germination to 48 h (PO-48 h) significantly enhanced the desirable aromas in peanut oil including nutty (5.6 ± 0.3), roasted (5.8 ± 0.1), butter (3.8 ± 0.2), and sweet notes (4.4 ± 0.2). However, it also generated moderate burnt (4.3 ± 0.1) and smoky odors (3.1 ± 0.3), which might disrupt the overall aroma profile of peanut oil.

The enhanced aroma intensity may be linked to increased free amino acid and reducing sugar contents during germination, which likely act as key precursors for Maillard reactions and lipid oxidation during roasting, thereby contributing to roasted and nutty aromas (Hu et al., 2025). Germination exhibited a strong positive correlation with roasted (*r* = 0.99), sweet (*r* = 0.98), and nutty (r = 0.98) attributes (*p* < 0.01), and a strong negative correlation with fatty (*r* = −0.97) and green (*r* = −0.98) aromas (*p* < 0.01), a trend consistent with its negative association with total aldehydes (*r* = −0.96, *p* < 0.01). Additionally, reducing sugars and free amino acids showed strong positive correlations with roasted (r = 0.98 and 0.96), sweet (*r* = 0.97 and 0.96), and nutty (r = 0.99 and 0.97) aromas, respectively (*p* < 0.01). Collectively, variations in the sensory quality of germinated peanut oils are likely attributed to compositional changes during germination, which are further modulated by microwave roasting and the relative concentrations of key volatile compounds. These findings suggest that germination pretreatment may serve as a viable strategy for producing peanut oil with improved sensory profiles that are more preferred by consumers.

## 4 Conclusion

This study investigated the effects of short-term peanut germination prior to microwave roasting on the flavor and oxidation stability of hydraulic-extracted peanut oil. Appropriate germination activated endogenous amylase, lipase, and proteases, promoting macronutrient hydrolysis and increasing free amino acids and reducing sugars (key Maillard reaction precursors). It elevated relative concentrations of pyrazines and volatile phenolics, reduced total aldehydes, and enhanced desirable nutty, roasted, butter, and sweet sensory notes, though over-germination (48 h) induced excessive undesirable smoky and burnt aromas. Germination also increased α- and β-tocopherol contents and extended oxidation induction time, indicating improved oxidative stability of peanut oil. These findings support short-term germination as a mild pretreatment to modulate roasted peanut oil flavor and stability. Future research should optimize germination conditions, explore endogenous enzyme regulation mechanisms, and characterize aroma-active compounds via molecular sensory techniques to establish a basis for precise peanut oil flavor modulation by germination.

## CRediT authorship contribution statement

**Gul Nawaz:** Writing – original draft, Investigation, Formal analysis, Data curation. **Wen-ting Yin:** Writing – review & editing, Supervision, Conceptualization. **Guang-yong Qin:** Supervision. **Hua-min Liu:** Funding acquisition. **Hui-li Pang:** Formal analysis. **Fan Zhang:** Methodology. **Qing-qing Ruan:** Data curation. **Zhi Qin:** Methodology. **Jun-ru Li:** Methodology.

## Declaration of competing interest

The authors declare that they have no known competing financial interests or personal relationships that could have appeared to influence the work reported in this paper.

## Data Availability

Data will be made available on request.
